# Tea plant-inspired nanoassembled supraparticles alleviate colitis and associated mental disorders via microbiota-gut-brain interactions

**DOI:** 10.7150/thno.113573

**Published:** 2025-06-18

**Authors:** Qinling Liu, Yunxiang He, Qingxin Fan, Yue Wu, Jiawen Li, Siqi Deng, Qiuping Xie, Yueling Zhao, Junling Guo, Xiao Du

**Affiliations:** 1 Tea Resources Utilization and Quality Testing Key Laboratory of Sichuan Province, College of Horticulture, Sichuan Agricultural University, Chengdu, Sichuan 611130, China.; 2 BMI Center for Biomass Materials and Nanointerfaces, National Engineering Laboratory for Clean Technology of Leather Manufacture, Ministry of Education Key Laboratory of Leather Chemistry and Engineering, College of Biomass Science and Engineering, Sichuan University, Chengdu, Sichuan 610065, China.; 3 Department of Orthopedics, The First Affiliated Hospital of Chongqing Medical University, Chongqing Medical University, Yuzhong, Chongqing 400016, China.; 4 Key Laboratory of Birth Defects and Related Diseases of Women and Children of MOE, Department of Pediatrics, West China Second University Hospital, Sichuan University, Chengdu, Sichuan 610041, China.; 5 State Key Laboratory of Polymer Materials Engineering, Sichuan University, Chengdu, Sichuan 610065, China.; 6 Bioproducts Institute, Department of Chemical and Biological Engineering, The University of British Columbia, Vancouver, BC V6T 1Z4, Canada.

**Keywords:** EGCG, tea proteins, supraparticles, inflammatory bowel disease, microbiota-gut-brain interactions

## Abstract

**Background:** Inflammatory bowel disease (IBD) is a chronic inflammatory condition of the gastrointestinal tract that significantly impacts patient health and quality of life. (-)-Epigallocatechin-3-gallate (EGCG), a potent plant polyphenol from green tea, exhibits superior anti-inflammatory and antioxidative properties; however, its therapeutic potential is hindered by poor stability and bioavailability.

**Methods and results:** Inspired by the tea plant (*Camellia sinensis*), we developed EGCG-loaded tea supraparticles (TSPs) as an oral dietary supplement, utilizing tea proteins, an eco-friendly byproduct with inherent antioxidative potential, to deliver EGCG. TSPs greatly improved EGCG's stability during gastrointestinal transport, preserving its antioxidant properties and its ability to modulate the immune microenvironment. In a dextran sulfate sodium salt-induced colitis mouse model, TSPs treatment reduced the disease activity index by more than 70% and showed a 1.53-fold improvement in efficacy than EGCG alone. Enhanced colonic barrier integrity and anti-inflammatory effects were observed by oral administration of TSPs. Furthermore, TSPs modulated gut microbiota, promoting microbial diversity and homeostasis thereby alleviating systemic inflammation. This reduction in inflammation contributed to improved blood-brain barrier integrity, potentially mitigating anxiety and depressive-like behaviors associated with colitis.

**Conclusion:** These findings highlight the potential of TSPs as a sustainable nanotechnology-based strategy for enhancing the efficacy of EGCG and effectively addressing IBD and its associated complications.

## Introduction

Inflammatory bowel disease (IBD), encompassing Crohn's disease and ulcerative colitis, is a chronic gastrointestinal disorder affecting over 3.5 million people, with rising prevalence rates worldwide 1, 2. The pathogenesis of IBD is driven by a complex interplay between genetic, environmental, and immunological factors, with oxidative stress and gut microbiota playing central roles in driving inflammation and disease progression 3-5. Microbial imbalance impairs immune homeostasis and compromises intestinal barrier function. Oxidative stress causes the infiltration of immune cells such as neutrophils and macrophages into the gut mucosa, triggering the production of reactive oxygen species (ROS) and inflammatory cytokines, which exacerbate tissue damage 6, 7. Modulation of inflammation and gut microbiota remains an effective strategy for IBD management 8, 9.

The impact of IBD often extends beyond the gut, contributing to systemic inflammation and increased permeability of the blood-brain barrier (BBB) 10-13. This systemic response is linked to neuropsychiatric comorbidities such as anxiety and depression, affecting up to 35% of patients, and is thought to be driven by disruptions in microbiota-gut-brain axis 14-17. Current treatments often fail to achieve sustained remission and are accompanied by significant side effects, highlighting the need for more effective therapeutic strategies that target both gut inflammation and its systemic consequences.

(-)-Epigallocatechin-3-gallate (EGCG), a plant polyphenol derived from green tea, exhibits well-documented anti-inflammatory, antioxidant, and gut-modulating properties, making it a promising therapeutic agent for IBD 18, 19. However, its clinical utility is hindered by poor stability and limited bioavailability. To overcome these challenges, nanotechnology-based delivery systems, such as liposomes, nanomicelles, and polymeric nanoparticles, have been explored 20-22, offering improved solubility, controlled release, and enhanced tissue targeting 23-25. Despite these benefits, synthetic nanocarriers often raise concerns regarding fabrication complexity and long-term safety. As a natural alternative, plant-derived proteins offer favorable biocompatibility, biodegradability, and functional adaptability 26, 27. Protein-polyphenol assemblies, formed through non-covalent interactions, offer a simple preparation process, and excellent biocompatibility, distinguishing them from synthetic nanocarriers 28-30.

In the tea plant (*Camellia sinensis*), tea polyphenol (~20% of leaf mass) naturally coexists with tea proteins (TProtein, also ~20% of leaf mass) 31, 32, likely forming self-assembled complexes through supramolecular interactions to involve in plant defense and tolerance. Inspired by this naturally evolved synergy, we engineered EGCG-TProtein complexes that mimic these native interactions to enhance EGCG's functionality.

**Figure 1:**
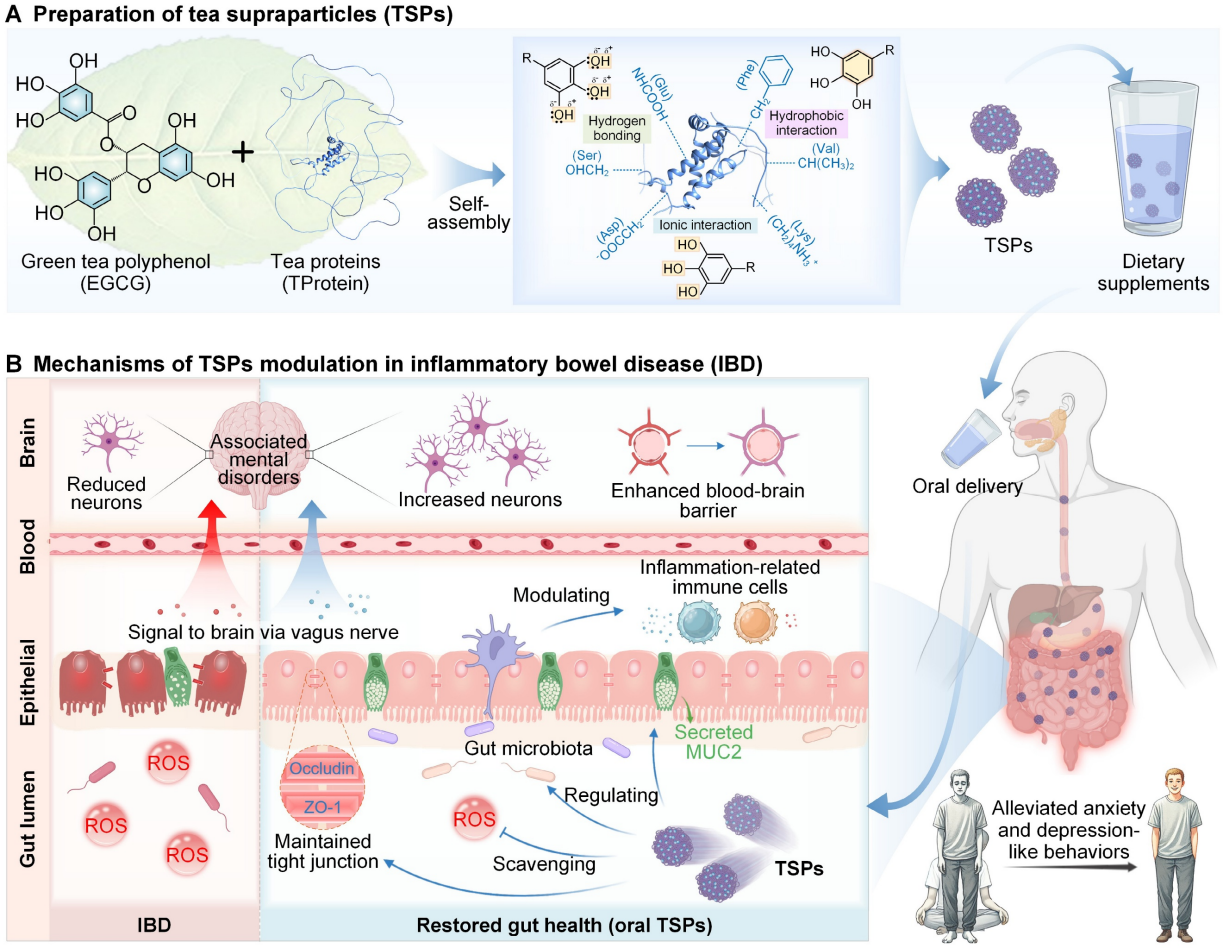
** Preparation of TSPs and their modulatory effects on IBD and associated mental disorders. (A)** EGCG and TProtein nanoassembled into TSPs through supramolecular interactions, as an oral dietary supplement. **(B)** Mechanisms of TSPs for restoring gut health and associated mental disorders through the microbiota-gut-brain axis.

In this study, we developed EGCG-loaded tea supraparticles (TSPs) as an oral dietary supplement and evaluated the therapeutic efficacy in a dextran sulfate sodium salt (DSS)-induced colitis mouse model (**Figure 1**). TSPs significantly improved EGCG stability during gastrointestinal transit, effectively preserving its antioxidative properties. In the colitis model, oral TSPs reduced disease activity index (DAI) scores by over 70%, compared to 45% in the EGCG-only group. Our findings suggest that TSPs promoted gut health by modulating the colonic mucosal layer, restoring epithelial barrier function, and reshaping gut microbiota composition. Furthermore, TSPs reduced both local and systemic inflammation, potentially alleviating IBD-associated neuroinflammation, as evidenced by behavioral tests showing reductions in anxiety and depressive-like behaviors. This bioinspired, safe and natural dietary therapeutic strategy offers a promising strategy for alleviating colitis and associated mental disorders via microbiota-gut-brain interactions.

## Results

### Design and characterization of TSPs

As a sustainable byproduct from tea leaves, primarily consisting of glutelins and prolamins, TProtein demonstrates biofunctional properties, including antioxidation 31-33. To assess the impact of the alkaline extraction on TProtein, we performed Fourier transform infrared (FTIR) spectroscopy. Analysis of the amide I band (1700-1600 cm⁻¹) showed minimal changes in the secondary structure, with the TProtein retaining a structure similar to that of the original tea leaf proteins (**Table S1**). The average molecular weight of the extracted TProtein was 47 kDa by quantitative proteomics.

**Figure 2:**
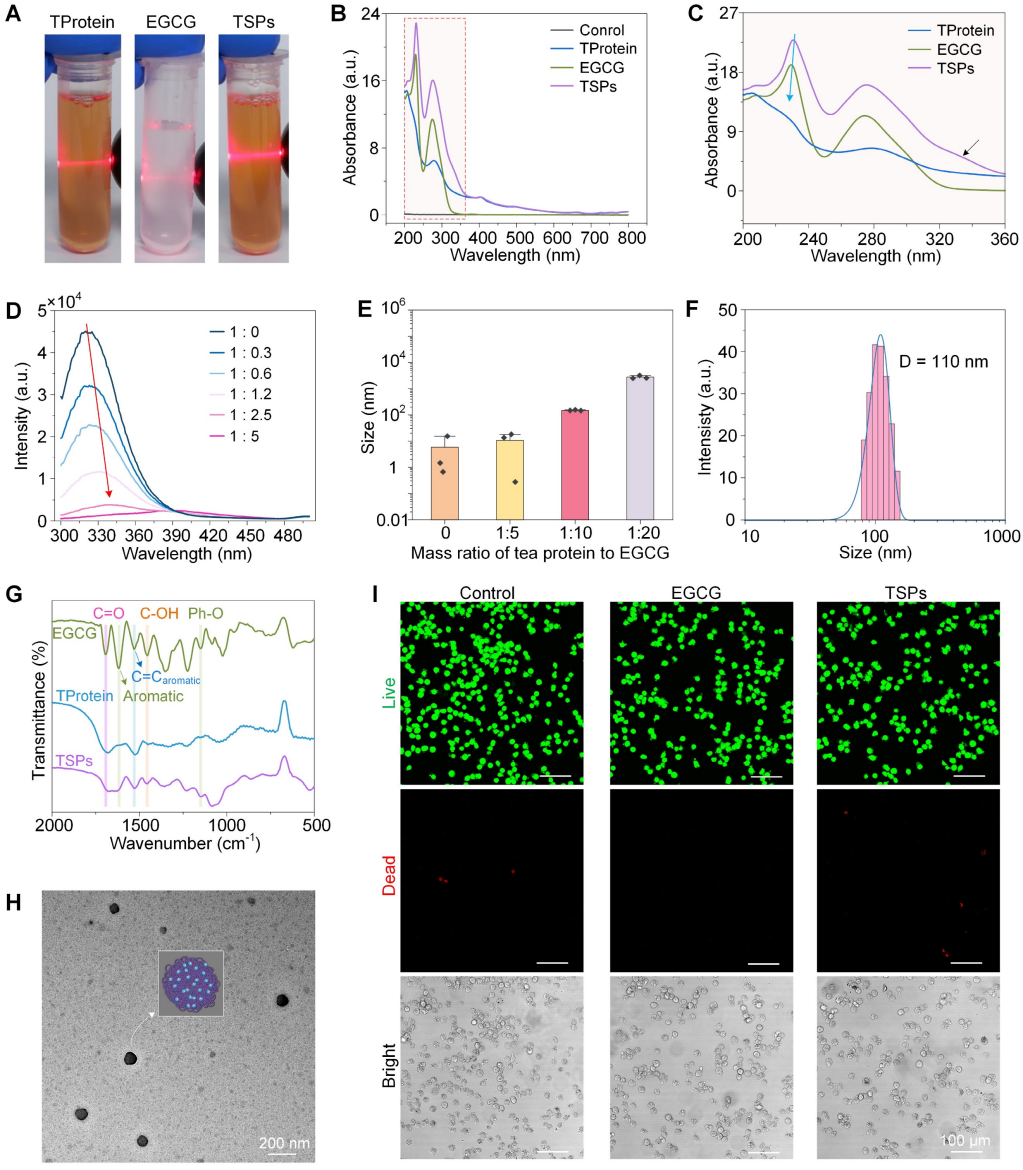
** Design and characterization of TSPs. (A)** Photographic images of TSPs solutions under laser irradiation. **(B)** UV-Vis spectra of the samples.** (C)** Magnified UV-Vis spectra highlighting the characteristics of TSPs. **(D)** Fluorescence spectra showing changes with different mass ratios of TProtein to EGCG at 25 °C. **(E)** Supraparticle sizes at different mass ratios of TProtein to EGCG. **(F)** The size distribution of the nanoparticles was measured by DLS. **(G)** FTIR spectra of the samples. **(H)** TEM image of the nanoparticles. **(I)** Live/dead fluorescence imaging of L929 cells treated with the supraparticles for 24 h.

TSPs were prepared through the self-assembly of TProtein and EGCG into supraparticles without chemical cross-linking agents or organic solvents under a cell-friendly condition (in phosphate buffered saline, PBS solution, pH 7.6). The stronger light scattering (**Figure 2A**) observed in the TSPs solution, compared with TProtein alone, suggests the formation of larger aggregates. The interaction between EGCG and TProtein was further confirmed by the blue shift and the emergence of a new shoulder peak in the ultraviolet-visible spectroscopy (UV-vis) (**Figure 2B**-**C**), reflecting changes in the optical properties of the components following their assembly. With increasing EGCG concentration, the fluorescence emission spectrum of TProtein broadened and red-shifted, suggesting the interaction between EGCG and TProtein (**Figure 2D**). Fluorescence quenching was observed, likely due to the disruption of the spatial arrangement of chromophores, such as tryptophan, tyrosine, and phenylalanine, upon binding with EGCG. The mass ratio of TProtein and EGCG was optimized to achieve stable and appropriately sized supraparticles (**Figure 2E**). The 1 : 10 ratio was identified as the most effective, producing supraparticles with a uniform size of approximately 110.20 ± 0.48 nm, as measured by dynamic light scattering (DLS) (**Figure 2F**). The FTIR spectra (**Figure 2G**) showed the phenolic characteristic peaks of C=O (1690 cm⁻^1^), aromatic (1620 cm⁻^1^), C=C_aromatic_ (1528 cm⁻^1^), C-OH (1455 cm⁻^1^), and Ph-O (1149 cm⁻^1^), confirming the successful nanoassembly of EGCG into the TSPs. The characteristic peak of EGCG at 1690 cm⁻¹ shifted to 1677 cm⁻¹ in TSPs, while the TProtein peak at 1524 cm⁻¹ red-shifted to 1529 cm⁻¹ in TSPs, indicating potential hydrogen bonding and hydrophobic interactions between EGCG and TProtein. Moreover, the C=C_aromatic_ peak of EGCG at 1620 cm⁻¹ shifted to 1625 cm⁻¹ in TSPs, which could be attributed to the π-π stacking interactions between EGCG and TProtein. Collectively, the spectral shifts confirm the successful assembly of EGCG and TProtein into a stable supramolecular structure through multiple non-covalent interactions.

The transmission electron microscope (TEM) image (**Figure 2H**) further validated the findings, showing uniformly sized spherical particles with an average diameter of approximately 100 nm. The hydrophilicity of TSPs was slightly lower than EGCG (**Figure S1**), and the zeta potential of TSPs was similar to EGCG but more negative than that of TProtein (**Figure S2**). Moreover, biocompatibility was assessed using live/dead fluorescence imaging (**Figure 2I**). The results showed no cytotoxicity in L929 cells treated with TSPs after 24 h, similar to the untreated control and EGCG-treated cells. Overall, the assembly and characterization of TSPs were demonstrated, achieving optimal size, stability, and biocompatibility under specific conditions.

### EGCG stability and functionality enhanced by TSPs

**Figure 3:**
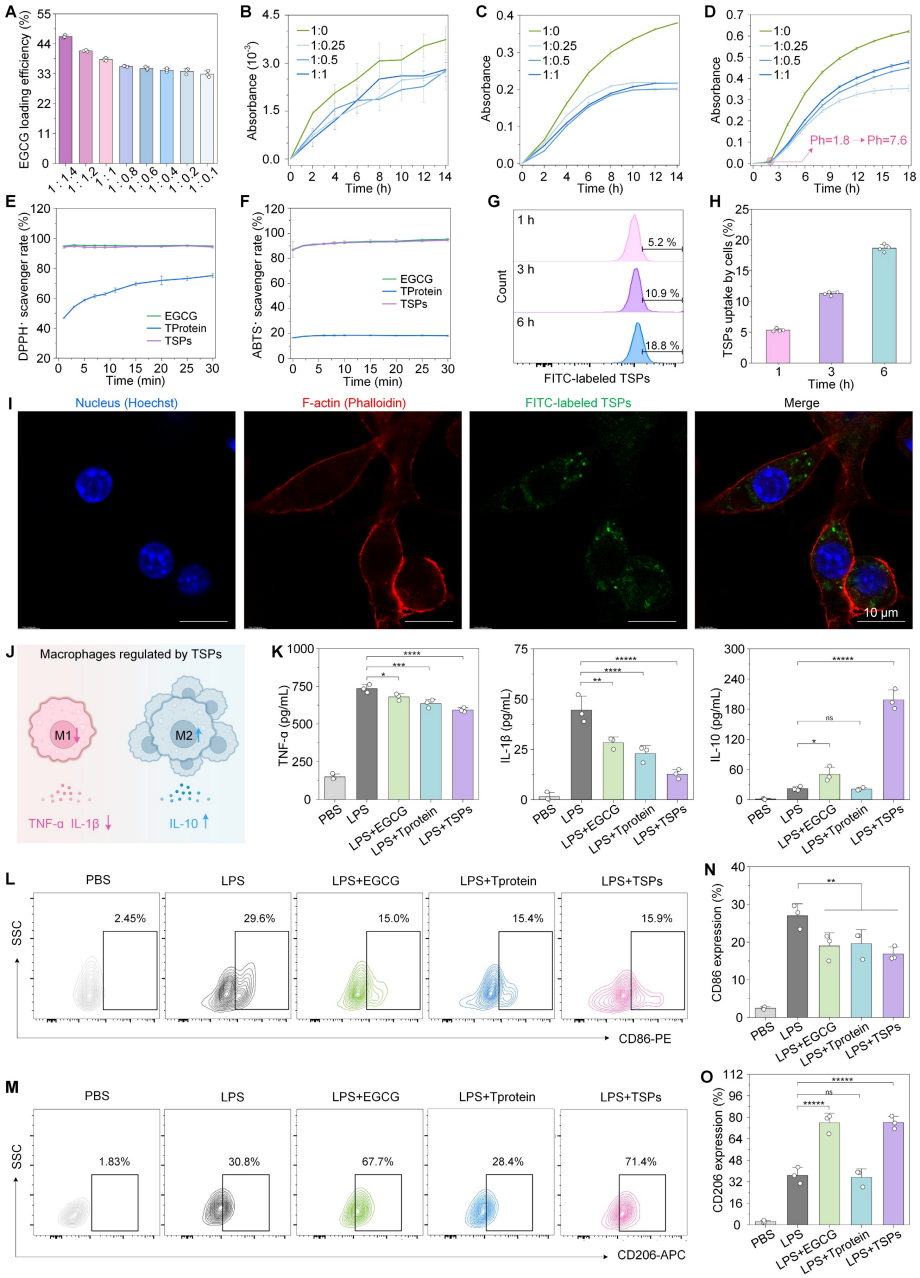
**Enhanced stability and antioxidative activity of TSPs. (A)** EGCG loading efficiency (%) of TSPs at various EGCG-to-TProtein mass ratios. **(B)** EGCG oxidation kinetics at gastric pH (1.8) with different EGCG-to-TProtein mass ratios. **(C)** EGCG oxidation kinetics at intestinal pH (7.6). **(D)** EGCG oxidation under sequential pH conditions simulating gastrointestinal transition. **(E-F)** DPPH· and ABTS· free radical scavenging activity over time. **(G)** Proportion of EGCG uptake by cells analyzed by flow cytometry after co-incubation for 1, 3, and 6 h. **(H)** Quantification of the positive proportion of EGCG uptake by cells. **(I)** Uptake profiles by L929 cells of TSPs at 6 h. **(J)** Schematic representation of macrophage regulation by TSPs. **(K)** *In vitro* anti-inflammatory activities of EGCG, TProtein, and TSPs. **(L-M)** Representative flow cytometry plots of CD86 and CD206 cells in RAW264.7 macrophages upon stimulation with LPS. **(N-O)** Positive ratio of CD86 and CD206 macrophages in RAW264.7 cells. Statistical significance was determined using one-way Analysis of Variance (ANOVA) followed by a least significant difference (LSD) post-hoc test. ns > 0.05, * P < 0.05, ** P < 0.01, *** P < 0.001, **** P < 0.0001, and *****P < 0.00001.

Higher protein content resulted in improved loading efficiency, reaching up to ~50% at an EGCG-to-TProtein ratio of 1:1.4 (**Figure 3A**). To assess the protective effect of TSPs on EGCG during gastrointestinal transit, we accessed EGCG stability at different pH levels by tracking absorbance at 425 nm 34. Under acidic conditions (simulated gastric pH = 1.8), TSPs reduced EGCG oxidation by approximately 30% compared with EGCG alone (**Figure 3B**). Additionally, TSPs show certain stability under simulated gastric fluid (**Figure S3**). Under alkalescent conditions (simulated intestinal pH=7.6), EGCG oxidized more rapidly, with absorbance increasing from 0 to 0.38 over 14 h (**Figure 3C**). In contrast, the oxidation rate of TSPs decreased by nearly half, and the absorbance only rose to 0.20. Furthermore, under sequential pH changes from acidic to alkalescent, TSPs significantly mitigated EGCG oxidation compared with free EGCG (**Figure 3D**). These findings demonstrate that TSPs effectively protect EGCG from oxidative degradation under physiologically relevant pH environments.

To assess the antioxidant performance of TSPs, 1,1-diphenyl-2-picryl-hydrazyl (DPPH·) (**Figure 3E**) and 2,2'-azino-bis (3-ethylbenzothiazoline-6-sulfonic acid) (ABTS·) (**Figure 3F**) free radical scavenging assays were conducted. The results showed that the free radical scavenging activity of TSPs was comparable to that of free EGCG over the 30-min measurement period. In contrast, TProtein alone showed negligible activity.

Most nanoparticle carriers can enter intestinal cells directly through clathrin-mediated endocytosis, caveolae-mediated endocytosis, or micropinocytosis 35. Based on this, we evaluated the cellular uptake of TSPs. Flow cytometry analysis (**Figure 3G, H**) revealed a time-dependent increase in intracellular fluorescence intensity in TSPs-treated cells, indicating progressive uptake over the 6 h. This was further confirmed by confocal microscopy (**Figure 3I**), where distinct green fluorescence inside the cells demonstrated successful EGCG delivery via TSPs.

In IBD, macrophage activation by ROS, and pathogen- or damage-associated molecular patterns trigger NF-κB and PI3K/AKT signaling, upregulating pro-inflammatory cytokines and enzymes, which drive oxidative damage in healthy tissues 36. To evaluate the immunomodulatory effects of TSPs, we measured key cytokine secretion in RAW 264.7 macrophages following LPS stimulation (**Figure 3J**). The results showed that TSPs effectively reduced the secretion of pro-inflammatory cytokines, including TNF-α and IL-1β, compared to both EGCG and TProtein alone (**Figure 3K**). TSPs significantly increased the secretion of the anti-inflammatory cytokine IL-10 to 198.55 ± 16.30 pg/mL, which was significantly higher than the levels observed with EGCG (50.25 ± 11.95 pg/mL) and TProtein (21.12 ± 2.30 pg/mL) alone. Flow cytometry analysis revealed that TSPs significantly reduced the percentage of CD86+ (M1) macrophages and increased CD206+ (M2) macrophages (**Figure 3L-O**). This shift was accompanied by changes in cytokine levels, suggesting that TSPs promote M2 polarization by triggering key signaling pathways 37.

### Enhanced therapeutic efficacy of TSPs in mitigating DSS-induced colitis

**Figure 4:**
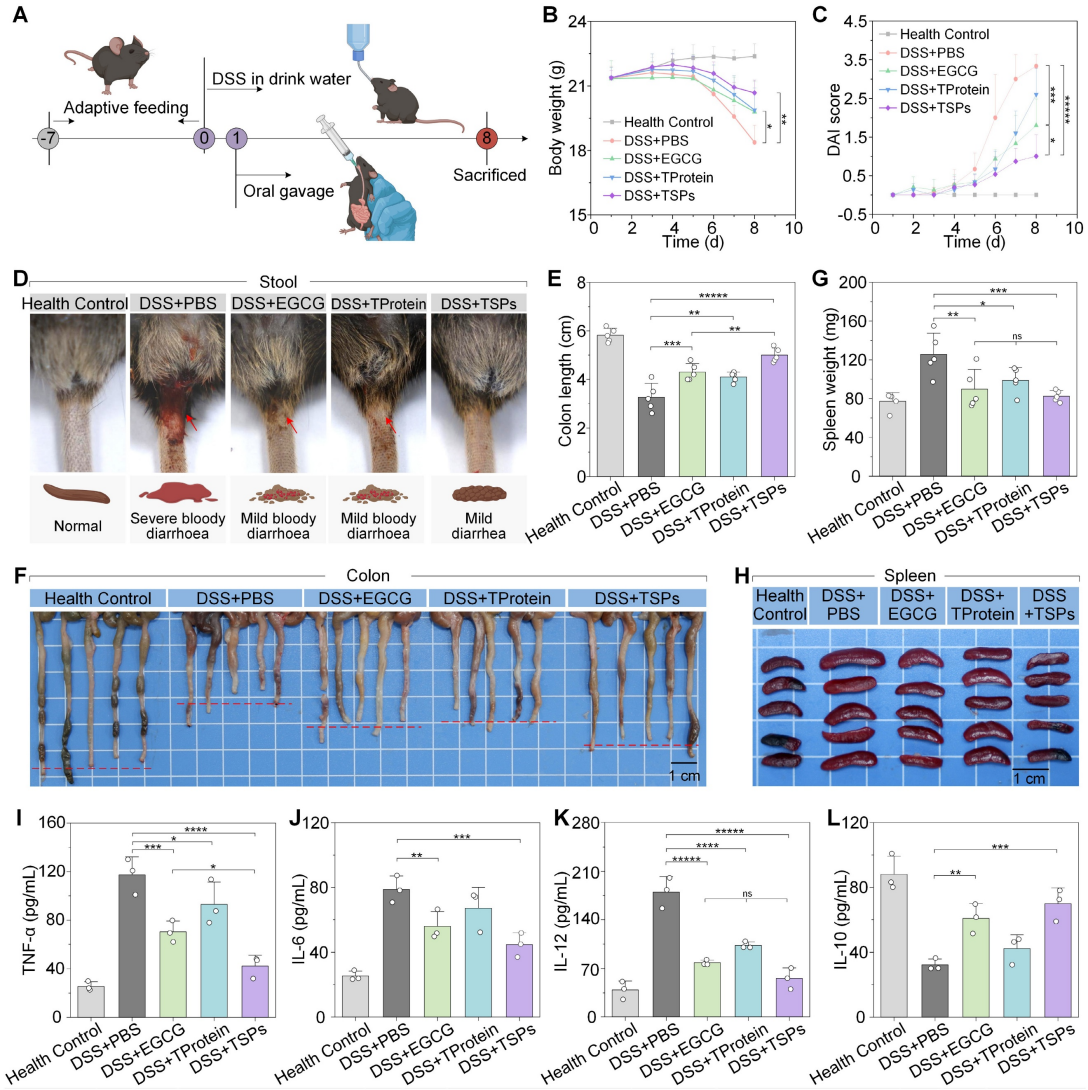
** Therapeutic effects of different treatments on DSS-induced colitis. (A)** Schematic representation of the experimental protocol. **(B)** Changes in body weight over the experimental period. **(C)** DAI scores reflect colitis severity. **(D)** Representative images of stool consistency and rectal bleeding across treatment groups. **(E)** Colon length as a marker of colitis severity. **(F)** Representative images of colons from each treatment group. **(G)** Spleen weight as an indicator of systemic inflammation. **(H)** Representative images of spleens from each treatment group. **(I-L)** Cytokine levels in serum were measured by ELISA. Data are presented as means ± SD (n = 5 in B, C, E, and G; n = 3 in I-L). Statistical significance was determined using one-way ANOVA followed by an LSD post-hoc test. ns > 0.05, * P < 0.05, ** P < 0.01, *** P < 0.001, **** P < 0.0001, and *****P < 0.00001.

The therapeutic effects of each treatment on DSS-induced colitis were systematically evaluated (**Figure 4A**). Over the 7-d observation period, mice in the DSS + PBS group progressively developed chronic colitis, characterized by continuous body weight loss, diarrhea, and hematochezia. On the final day, mice in the DSS + PBS group had lost nearly 15% of their initial body weight, with DAI scores up to 3.3 ± 0.3 (**Figure 4B**-**C**). DAI scores in the DSS + EGCG group dropped to 1.8 ± 0.7, while in the DSS + TSPs group were only 1.0 ± 0.6, close to that of healthy mice. The DSS + TSPs group effectively alleviated diarrhea and hematochezia (**Figure 4D**), while effectively restoring the levels of red blood cells, hemoglobin and hematocrit, thereby mitigating DSS-induced iron-deficiency anemia (**Figure S4**).

Colon length, a critical marker of colitis severity, was evaluated 38. In the DSS + PBS group, colons appeared significantly shortened with visible hemorrhagic areas, indicating extensive inflammation and epithelial damage (**Figure 4F**). DSS exposure reduced colon length to approximately 60% of the health control group, while the colon in the DSS + TSPs group restored it to over 90%, achieving a 2-fold improvement compared with other therapeutic groups (**Figure 4E**). Spleen enlargement is often a sign of systemic immune activation. Spleen images (**Figure 4H**) revealed marked enlargement in the DSS + PBS group, reflecting pronounced systemic inflammation. Compared to the other treatment groups, spleen weight and spleen index (**Figure 4G** and** S5**) in the DSS + TSPs group were closest to normal levels, suggesting reduced immune activation associated with colitis.

Serum concentration of pro-inflammatory cytokines, increases BBB permeability, promoting neuroinflammation and impairing neural function 12, 13. Mice in the DSS + EGCG group and the DSS + TProtein group showed a reduction in pro-inflammatory cytokine (TNF-α, IL-6, and IL-12) levels by approximately 30-60% (**Figure 4I-K**). The pro-inflammatory cytokine levels in the DSS + TSPs group decreased by nearly 40-70%. Additionally, the anti-inflammatory cytokine IL-10 showed the opposite trend (**Figure 4L**). Furthermore, TSPs treatment restored white blood cell counts and lymphocyte percentages to near-normal levels (**Figure S6**). These findings preliminarily suggest that TSPs hold strong therapeutic potential for reducing systemic inflammation and restoring BBB integrity.

### TSPs restore colonic and systemic health in DSS-induced colitis

**Figure 5:**
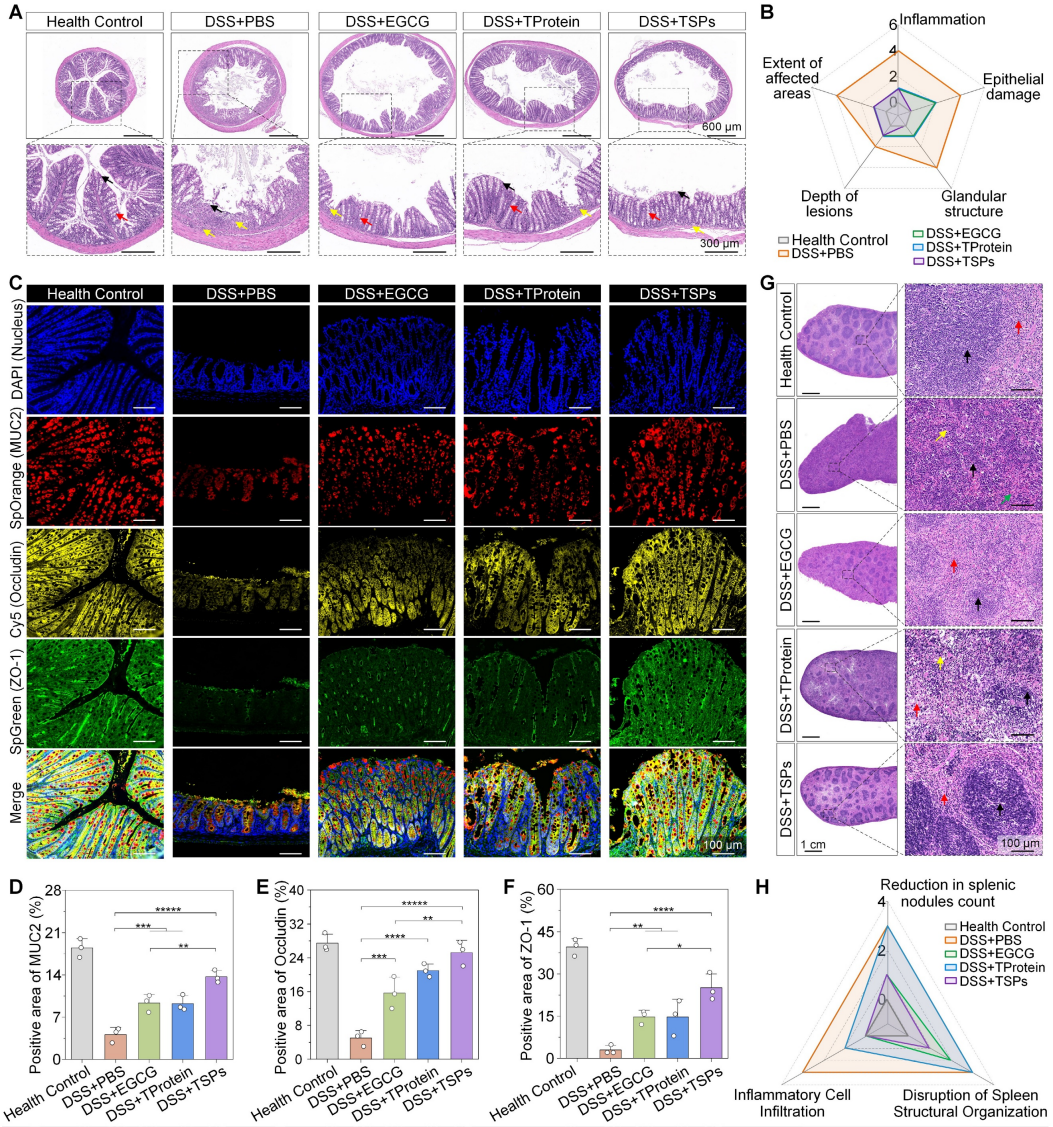
** Histological and immunofluorescence analysis of colonic and splenic tissues. (A)** Representative Hematoxylin and Eosin (H&E) staining images of colonic tissues. Red arrows: goblet cells. Black arrows: epithelial cell. Yellow arrows: areas of inflammatory cell infiltration. **(B)** Radar chart illustrating histopathological scoring parameters of the colon, with overlapping results for the 'DSS+TProtein' and 'DSS+EGCG' groups. **(C)** Immunofluorescence staining of colonic tissues. **(D-F)** Quantitative analysis of positive areas for MUC2, Occludin, and ZO-1 expression. Data are presented as means ± SD (n = 3). **(G)** H&E staining of spleen tissues. Black arrows: splenic white pulp spleen nodules. Red arrows: splenic red pulp. Yellow arrows: multinucleated macrophage. Green arrows: neutrophil hyperplasia. **(H)** Radar chart summarizing spleen histopathological scores. Statistical significance was determined using one-way ANOVA followed by an LSD post-hoc test. * P < 0.05, ** P < 0.01, *** P < 0.001, **** P < 0.0001, and ***** P < 0.00001.

Colonic histology and immunofluorescence were analyzed to evaluate the efficacy of different treatments in DSS-induced colitis. The DSS + PBS group exhibited severe intestinal damage, characterized by extensive mucosal ulceration and epithelial erosion, exposing the lamina propria (**Figure 5A**). The DSS + TSPs group demonstrated the most integrated tissue structure and minimal inflammatory cell infiltration. Furthermore, histopathological grading of colonic tissue sections was performed for quantitative comparison (**Figure 5B**).

Mucoprotein-2 (MUC2) is a key component of the intestinal chemical barrier, mainly secreted by goblet cells, and plays an essential role in maintaining gut health by forming a protective mucus layer 39. The recovery of MUC2 expression was most obvious in the DSS + TSPs group, with levels 1.83 times higher than those in the DSS + EGCG group (**Figure 5C**). Similarly, Occludin and zonula occludens-1 (ZO-1), essential tight junction proteins that regulate epithelial permeability 40, showed expression patterns consistent with MUC2 restoration (**Figure 5D-F**). These results demonstrate that oral TSPs restore both the chemical and mechanical gut barriers, safeguarding against bacterial invasion.

The spleen, essential for filtering pathogens and regulating immune responses, showed significant structural alterations following DSS administration (**Figure 5G**). The DSS + EGCG group showed moderate improvement with splenic nodules, increased lymphocyte counts, and occasional splenic nodule atrophy. Minimal efficacy was seen in the DSS + TProtein group. The DSS + TSPs group showed the most significant recovery, with an increased number of splenic nodules, well demarcated from the red pulp, and no significant inflammatory cell infiltration. Besides, we quantitatively confirmed the histological findings (**Figure 5H**). Importantly, the therapeutic effect of TSPs surpassed the combined effects of EGCG and TProtein, suggesting that TSPs may enhance the systemic absorption and bioavailability of EGCG. Pathohistological analysis of major organs also showed no significant side effects across all treatment groups, indicating the biosafety of oral dietary supplements (**Figure S7**).

### Gut microbiota analysis

**Figure 6:**
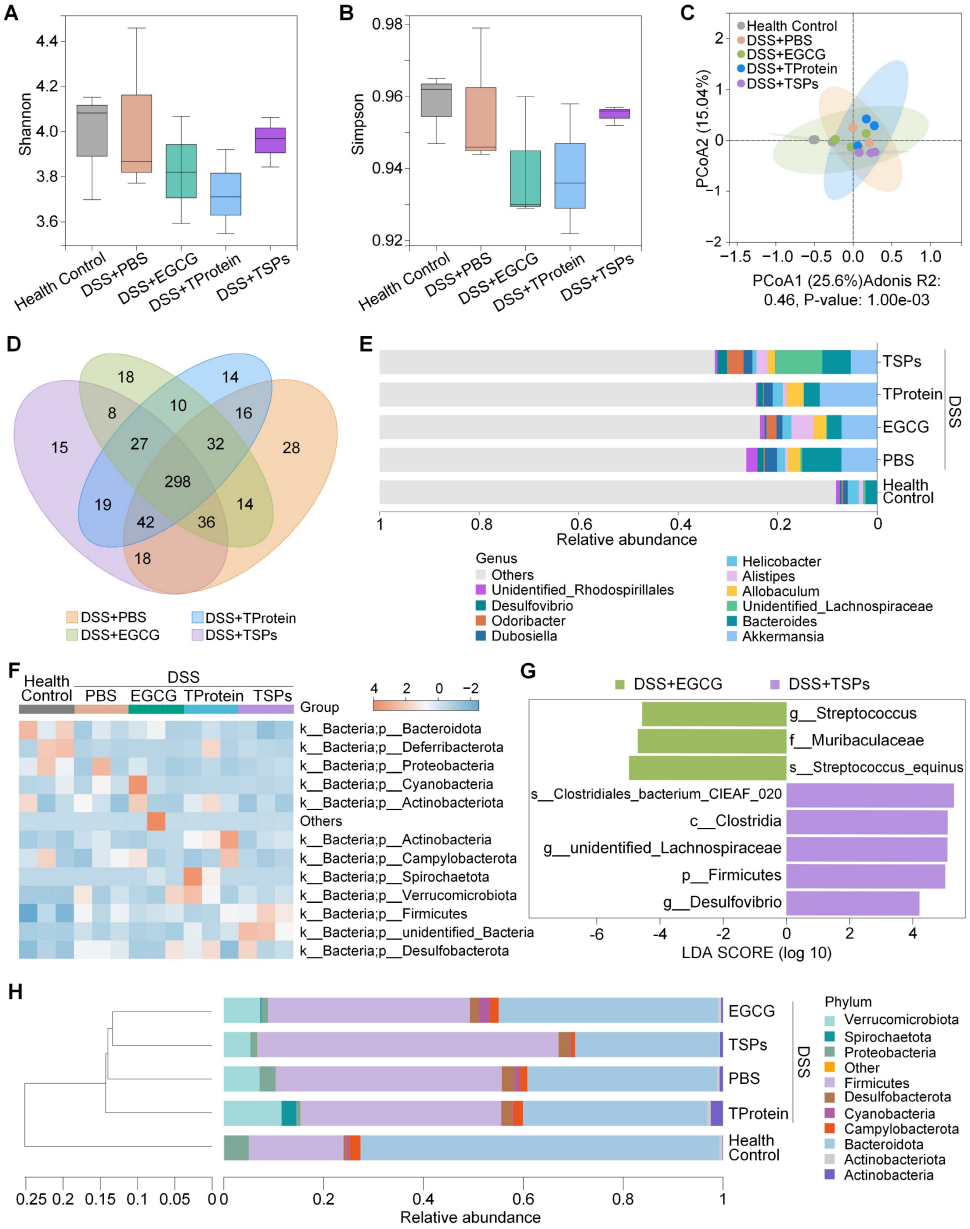
** TSPs regulate the composition of gut microbiota. (A)** Shannon index and **(B)** Simpson index reflecting α-diversity of the gut microbial community in each group. **(C)** PCoA showing β-diversity of the gut microbiota. **(D)** Venn diagram illustrating unique and shared operational taxonomic units among groups. **(E)** Stacked bar chart showing relative abundance at the genus level. Each column represents the mean of each group. **(F)** Heatmap displaying a relative abundance of flora at the phylum level. **(G)** LEfSe analysis identifying microbial biomarkers with significant differences between two groups. **(H)** UPGMA clustering tree showing microbial community similarity among groups. Each column represents the mean of each group. Data are presented as means ± SD (n = 3).

Growing evidence suggests that gut microbiota plays a critical role in regulating host mental health through the microbiota-gut-brain axis 41. To evaluate the regulatory effect of TSPs on the intestinal microbiota, 16S ribosomal DNA (16S rDNA) sequencing was conducted. Alpha diversity analysis (**Figure 6A-B**) demonstrated that the DSS + TSPs group restored microbial richness and evenness to levels comparable with the health control group. Principal Coordinate Analysis (PCoA, **Figure 6C**) demonstrated that the gut microbiota of mice treated with TSPs formed a distinct cluster compared to DSS-induced colitis mice. The Venn diagram (**Figure 6D**) illustrates shared and unique amplicon sequence variants (ASVs) among treatment groups.

Genus-level taxonomic analysis revealed significant changes in microbial composition among the treatment groups (**Figure 6E**). The DSS + PBS group exhibited a notable enrichment of pro-inflammatory microbes such as *Allobaculum*, which is known to aggravate colitis by promoting immune activation. The DSS + TSPs group effectively suppressed these harmful taxa while uniquely enriching beneficial genera like *Lachnospiraceae* and *Odoribacter* 42. These taxa are associated with short-chain fatty acid (SCFA) production and anti-inflammatory effects, contributing to gut barrier protection and systemic health. Conversely, the DSS + EGCG and DSS + TProtein group did not significantly enhance *Lachnospiraceae* abundance. In terms of microbial community profiles, the heatmap analysis provided a detailed comparison of microbial phyla across treatments (**Figure 6F**).

LEfSe (Linear discriminant analysis Effect Size) analysis (**Figure 6G**) was performed to identify microbial biomarkers with significant differences between the DSS + EGCG and DSS + TSPs groups. In the DSS + EGCG group, inflammatory-associated microbes such as *Streptococcus equinus* 43, 44 and *Streptococcus* persisted, indicating incomplete microbiota recovery. In contrast, the DSS + TSPs group exhibited a more pronounced microbiota-regulating effect. While small amounts of pro-inflammatory *Desulfovibrio* 45 were still detected, this group significantly enriched beneficial taxa such as *Clostridia* 46, *Lachnospira* 47, and *Firmicutes*. *Clostridia* 46, which produces butyrate, is crucial for gut health by maintaining barrier integrity and immune regulation. Moreover, *Lachnospira* is associated with myristic acid production, a fatty acid known to suppress neuroinflammatory cascades and support synaptic health 47. The results suggest that TSPs may help mitigate neuroinflammation and improve behavioral outcomes in IBD-related mental disorders. Despite taxonomic differences, UPGMA clustering (**Figure 6H**) showed similar overall microbial profiles between the DSS + EGCG and DSS + TSPs groups.

### Alleviation of colitis-associated mental disorders

**Figure 7:**
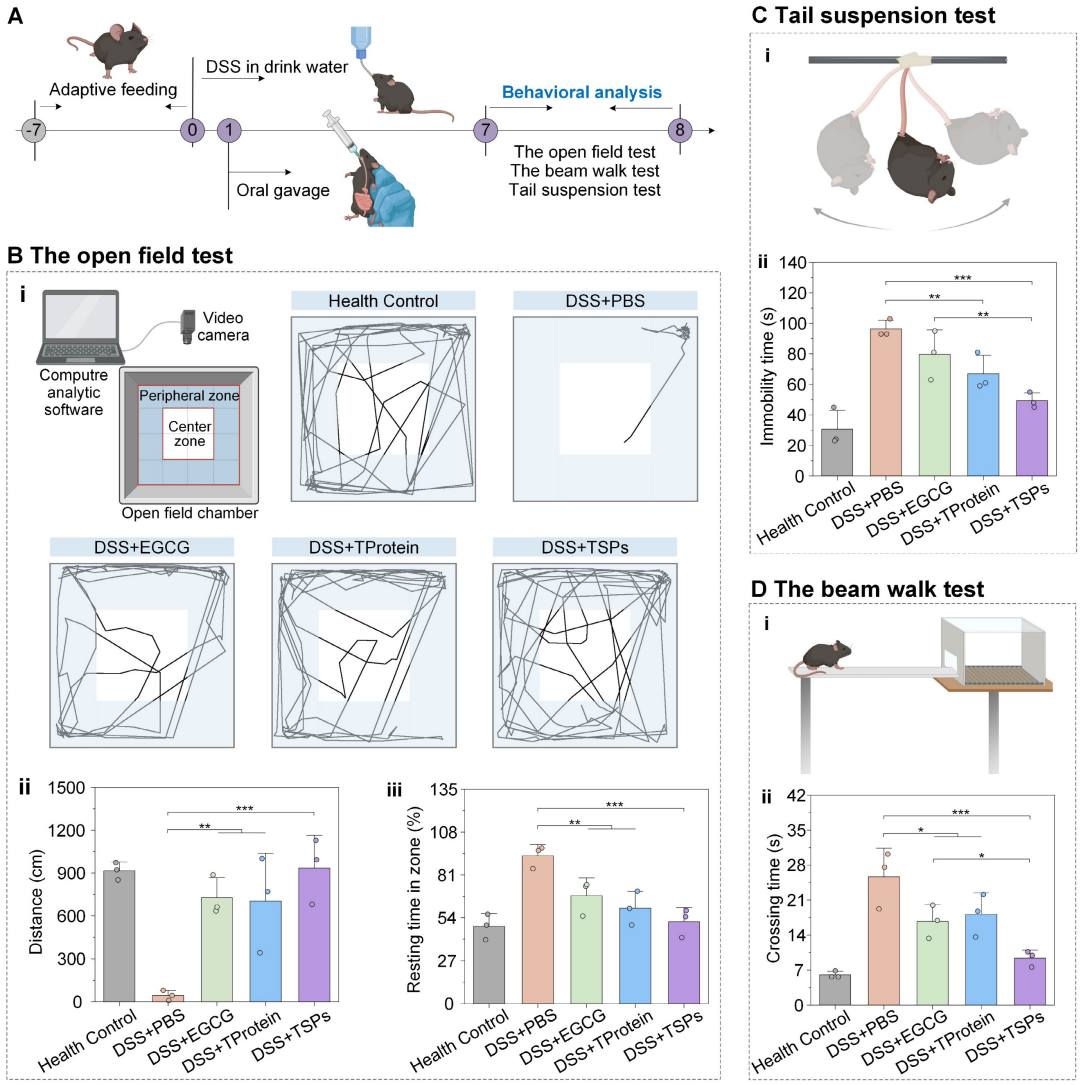
** Alleviation effects of TSPs on colitis-associated mental disorders. (A)** Experimental timeline and setup for DSS-induced colitis model and behavioral analysis. **(B)** The open field test: (i) Schematic diagram of the experiment and action roadmap results of mice, statistical results of total distance traveled (ii) and percentage of resting time (iii). **(C)** The tail suspension test. Schematic diagram (i) and statistical chart showing the immobility time (ii). **(D)** The beam walk test. Schematic diagram (i) and crossing times of mice across the beam (ii). Data are presented as means ± SD (n = 3). Statistical significance was determined using one-way ANOVA followed by an LSD post-hoc test. * P < 0.05, ** P < 0.01, *** P < 0.001, **** P < 0.0001, and ***** P < 0.00001.

Modulation of the gut microenvironment has been shown to improve autism spectrum disorder 48, antibiotic-associated anxiety 49, cognitive impairment 50, and athletic motivation 51. Behavioral tests were conducted on days 7-8 to evaluate the effects of treatment on colitis-associated mental disorders (**Figure 7A**). The open-field test trajectories (**Figure 7B-i**) showed that the DSS + PBS group exhibited highly restricted movement. The DSS + TSPs group displayed trajectories resembling the health control group, with balanced movement between peripheral and center zones, preliminarily indicating restored motor function and reduced anxiety. Mice in the healthy control group traveled an average of approximately 900 cm (**Figure 7B-ii**). In contrast, mice in the DSS + PBS group exhibited a dramatic reduction in total distance traveled, averaging less than 100 cm. DSS + TSPs group restored the total distance traveled to 934 cm, closely resembling health control group levels. Meanwhile, the longer resting time in the open field indicated higher levels of anxiety and less curiosity (**Figure 7B-iii**). The DSS + TSPs group showed the greatest improvement, reflecting alleviated anxiety and restored curiosity.

The tail suspension test (**Figure 7C-i**) assesses the depressive state by measuring the duration of immobility in mice. The DSS + PBS group showed a significant increase in immobility time to approximately 100 seconds, indicating severe behavioral despair (**Figure 7C-ii**). The DSS + EGCG group reduced immobility time to 80 seconds, while the DSS + TProtein group showed a similar reduction. Particularly, the DSS + TSPs group decreased immobility times to around 50 seconds, approaching the levels seen in the health control group.

The beam walk test (**Figure 7D-i**) assesses motor coordination and balance, which can be affected by systemic inflammation. Mice in the DSS +PBS group displayed severe motor deficits, taking approximately 25 seconds to cross the beam, 4-fold longer than that of the health control group (**Figure 7D-ii**). In contrast, mice in the DSS + TSPs group reduced crossing time to about 9 seconds, closely resembling the health control group. This highlights the ability of TSPs to reverse motor impairments more effectively than EGCG or TProtein alone.

## Discussion

In this study, we developed EGCG-loaded tea supraparticles (TSPs) as a novel nanotechnology-based strategy for mitigating the systemic effects of IBD. EGCG, a potent plant polyphenol, is well-known for its anti-inflammatory and antioxidant properties, with substantial therapeutic potential. Protein-based nanomaterials have been widely shown to exhibit longer half-lives, which can improve the pharmacokinetics of EGCG 52. Inspired by the tea plant, we incorporated TProtein as the delivery carrier of EGCG and utilized its natural antioxidative and biofunctional properties, offering an environmentally sustainable and cost-effective solution.

Inflamed mucosa exhibits an accumulation of positively charged proteins 53. Several studies have demonstrated that Nanoparticles with diameters under 200 nm and a negative surface charge showed enhanced tissue-penetration for IBD treatment, as these characteristics facilitate interactions with positively charged proteins on the damaged epithelium of IBD 54-56. TSPs, which are stable in the pH environment of the gut, maintain a nanoparticle form with a mean diameter of 110.20 ± 0.48 nm and a negative zeta potential. These properties enable TSPs to efficiently penetrate the intestinal mucus barrier and target inflamed regions of the colon. TSPs also protect EGCG from oxidative degradation under gastrointestinal pH conditions, which is due to the encapsulation of EGCG within TProtein, restricting molecular movement and providing steric shielding to reduce oxidation susceptibility 57. Furthermore, TSPs retain EGCG's ROS-scavenging capacity and effectively modulate gut immune responses.

Emerging evidence suggests that gut-derived inflammatory cytokines can translocate into the bloodstream through the damaged intestinal barrier, cross the BBB, and induce neuroinflammation 58. In our study, TSPs treatment enhanced intestinal barrier integrity, which likely limited the translocation of circulating cytokines to the brain and reduced central immune activation. This aligns with previous reports showing that improving gut barrier function can decrease hippocampal glial activation and neuronal injury, thereby alleviating IBD-associated mental disorders 59, 60. Moreover, the therapeutic effects of TSPs are closely linked to EGCG's capacity to modulate the gut microbiota. Fecal microbiota transplantation (FMT) from EGCG-treated mice into colitis recipients significantly improved DAI scores, enriched SCFA-producing bacteria, and alleviated both intestinal and systemic inflammation 18, 19. Whereas sterile fecal filtrates failed to reproduce these effects. These findings suggest that the brain-related benefits observed in TSP-treated mice may be partly attributed to EGCG's role in modulating neuroimmune pathways via microbiota-derived signaling. TSPs enriched several SCFA-producing genera, and SCFAs like butyrate have been shown to strengthen tight junctions in endothelial cells while reducing microglial activation, thus fostering a more stable neuroimmune environment 17, 19. In addition, *Lachnospiraceae* has been associated with the production of myristic acid, a fatty acid shown to inhibit neuroinflammatory cascades and support synaptic health 47. Given that the nucleus accumbens and hippocampus are particularly sensitive to inflammatory and metabolic cues 61, the modulation of peripheral inflammation and gut-derived metabolites by TSPs may translate into behavioral improvements via enhanced neuroplasticity.

Collectively, our findings suggest that TSPs exert systemic effects that extend beyond the gut, indirectly preserving BBB function and reducing central neuroinflammation, offering a promising approach to alleviate both gastrointestinal and neuropsychiatric symptoms in IBD. While both EGCG and TProtein are found in green tea, traditional tea consumption primarily extracts water-soluble compounds, leaving TProtein insoluble. Therefore, both components cannot be obtained simultaneously through tea drinking. The TSPs formulation, combining EGCG and TProtein, provides a synergistic approach, enhancing EGCG's therapeutic properties through improved delivery while TProtein serves as a biofunctional matrix for sustained release. As TProtein may differ slightly across cultivars and the extraction process is not yet fully standardized, some considerations for scalability and consistency remain. Moreover, this system has excellent biocompatibility. Both EGCG and TProtein are naturally derived plant extracts with no notable toxicity and are considered generally recognized as safe, which should facilitate their rapid translation into clinical applications.

## Material and Methods

### Extraction of TProtein

Green tea powder was mixed with 0.4 mol/L NaOH solution at a ratio of 1 : 25 (g/mL) and subjected to ultrasound-assisted extraction at 55°C, with an ultrasonic frequency of 500 W for 80 min. The resulting mixture was filtered through an 80-mesh gauze to remove tea residues. The filtrate was centrifuged at 4000 r/min for 20 min to collect the supernatant. Subsequently, the pH of the TProtein solution was adjusted to 4.5 using 1 mol/L HCl and maintained for 20 min. The mixture was then centrifuged again at 4000 r/min for 20 min to collect the precipitate. The precipitate was freeze-dried to obtain purified TProtein for further use. The Tprotein concentration of the extracted TProtein was quantified using the Coomassie Brilliant Blue assay, with a determined yield of 69%.

### Preparation of TProtein solution

A total of 1 mg of TProtein powder was weighed and dissolved in 6 mL of deionized water. The pH of the solution was adjusted to 12 using 1 mol/L NaOH to facilitate complete dissolution of the TProtein powder. Once fully dissolved, the pH was brought back to 7 using 1 mol/L HCl, and then the volume was fixed to 10 mL with PBS to obtain a TProtein solution with a final concentration of 0.1 mg/mL.

### Preparation of TSPs

TProtein solution (0.1 mg/mL) was mixed with EGCG solutions (purity 98%) at final concentrations of 0.1 mg/mL, respectively. The mixtures were shaken at 1400 rpm for 1 h at 25 °C in the dark to promote nanoparticle assembly. After standing, the solutions were illuminated with a laser pointer to confirm the presence of a distinct Tyndall effect, indicating the polymerization of TProtein into the larger-sized polymers. The solutions were then subjected to ultrasonic agitation at a frequency of 500 W for 5 min at room temperature. This process yielded nanoparticles with varying TProtein-to-EGCG ratios.

### Characterization of TSPs

To characterize the TSPs, the prepared solutions were first homogenized. A suitable volume of the solution was transferred to a plastic cuvette, respectively. After allowing the instrument to equilibrate for 120 seconds, the size of TSPs was measured.

Subsequently, the effect of EGCG on the intrinsic fluorescence intensity of TProtein was analyzed using a fluorescence spectrophotometer. Solutions with TProtein-to-EGCG mass concentration ratios of 1 : 0, 1 : 0.031, 1 : 0.625, 1 : 1.25, 1 : 2.5, and 1 : 5 were prepared, and 200 µL of each solution was transferred to a black 96-well plate. Fluorescence spectra for each sample were recorded using the spectrophotometer with an excitation wavelength of 250 nm and an emission wavelength range of 300-500 nm. The steady-state fluorescence measurements were conducted at a temperature of 25°C.

To analyze the optical properties of EGCG, TProtein, and TSPs, UV-Vis spectroscopy was performed using a microplate reader and transparent 96-well plates. Each sample was prepared by diluting to an appropriate concentration to avoid saturation. A 200 µL aliquot of each solution was added to the wells of the plate, and the absorbance spectra were recorded over a wavelength range of 200 nm to 800 nm.

The prepared TSPs were placed into a dialysis bag with a molecular weight cutoff of 300 kDa. Dialysis was performed against fresh dialysis buffers, which were periodically replaced to ensure the thorough removal of unbound components. After dialysis, the sample remaining inside the dialysis bag was collected and freeze-dried. A suitable amount of the freeze-dried sample was then mixed with potassium bromide (KBr) and pressed into pellets for FTIR spectroscopy analysis. The FTIR spectra were recorded over a scanning range of 4000 to 500 cm⁻¹.

### Determination of EGCG loading efficiency in TSPs

EGCG and TProtein were mixed at various mass ratios (EGCG : TProtein = 1:0.1 to 1:1.4) in PBS solution. The mixtures were vortexed briefly and then shaken at 1400 rpm for 1 h at room temperature in the dark to facilitate nanoparticle assembly. After incubation, the suspensions were transferred to 3 kDa ultrafiltration tubes and centrifuged to collect the filtrate. The concentration of EGCG in the filtrates was determined by high-performance liquid chromatography (HPLC). HPLC spectra were collected from the Agilent 1200 Series instrument (Agilent, USA). The separation was achieved from the CAPCELL PAK HPLC column (C18 250 mm × 4.6 mm, 5 µm). The mobile phase consisted of methanol and 0.1% phosphoric acid (32:68, v/v), with a flow rate of 1.0 mL/min. The column temperature was maintained at 25 °C, the injection volume was 20 μL, and detection was performed at 279 nm. The loading efficiency was calculated based on the difference between the initial and unbound EGCG concentrations.

### Assessment of EGCG stability in TSPs

To assess the oxidation stability of EGCG in the presence of TProtein, EGCG aqueous solutions (0.5 mg/mL) were mixed with TProtein at varying concentrations (0.25, 0.5, and 1 mg/mL). The pH of the mixtures was adjusted to either acidic (simulated gastric, pH 1.8) or alkaline (simulated intestinal, pH 7.6) conditions using 2 mol/L HCl or 2 mol/L NaOH buffer solutions, respectively. All samples were incubated at 30°C in the dark. The oxidative degradation of EGCG was monitored by measuring absorbance at 425 nm at 2-h intervals over 14 h. Additionally, to simulate the gastrointestinal pH transition, samples were first incubated at pH 1.8 for 2 h, then adjusted to pH 7.6 and further incubated for 16 h. Absorbance measurements were performed at 2-h intervals to assess the protective effect of TSPs during sequential pH changes.

### Evaluation of DPPH· and ABTS· free radical scavenging activity of TSPs

The DPPH· and ABTS· free radical scavenging activities of TSPs were determined using commercial DPPH· and ABTS· antioxidant assay kits (Solarbio, China). The tests were performed to evaluate the antioxidant capacity of the samples based on their ability to quench free radicals. The mass concentration ratio of EGCG (100 μg/mL) to TProtein was maintained at 10 : 1 in the preparation of TSPs.

### Cellular uptake assay of TSPs in L929 cells

L929 cells were cultured in DMEM without fetal bovine serum (FBS) for 12 h. Subsequently, the cells were incubated with FITC-labeled TSPs. To assess the cellular uptake efficiency, flow cytometry was performed at 1-, 3-, and 6-h post-incubation. The percentage of FITC-positive cells was measured using flow cytometry, which reflects the uptake efficiency of TSPs by the L929 cells.

### Macrophage polarization and cytokine profiles in RAW 264.7 macrophages

RAW 264.7 macrophages were seeded in 6-well plates at a density of 6 × 10^5^ cells per well, and LPS (200 ng/mL, final concentration) was added to each well to stimulate inflammation. At the same time, TSPs suspensions containing 30 μg/mL EGCG and 3 μg/mL TProtein (final concentration) were added to the wells. After a 24-h co-incubation period, the supernatants were collected, centrifuged at 3000 g for 10 min, and stored for cytokine analysis. The concentrations of various inflammatory cytokines in the supernatants were measured using enzyme-linked immunosorbent assay (ELISA) kits, according to the manufacturer's instructions (Servicebio, Wuhan, China).

To assess macrophage polarization, the macrophages from each well were collected. The macrophages were stained with Anti-Mouse CD86-PE and Anti-Mouse CD206-APC to identify M1 and M2 macrophages, respectively. The antibodies were sourced from Multi Sciences, Hangzhou, China. Flow cytometric analysis was performed with a NovoCyte (Agilent, California, USA).

### Animal experiments

Male C57BL/6 mice (6-8 weeks old, 19-22 g) were purchased from EnSiWeiEr Biotechnology Co., Ltd. (Chengdu, China). All mice were specific pathogen-free (SPF) grade and housed in a controlled environment (12-h light/12-h dark cycle, relative humidity 60%, temperature 24 ± 1°C) with *ad libitum* access to standard laboratory chow and sterile water. After a one-week acclimation period, the experiments were conducted following institutional guidelines and were approved by the Animal Care and Use Committee of West China Hospital, Sichuan University. No adverse events were observed during the study.

Mice were randomly divided into five groups (n = 5 per group): (1) Health control group: received sterile drinking water throughout the experiment; (2) DSS + PBS group: received 2.5% DSS (MW: 40000, Adamas-beta) in drinking water for 8 d; (3) DSS + EGCG Group: Received 2.5% DSS in drinking water and were gavaged with EGCG (25 mg/kg/d) starting on day 2; (4) DSS + TProtein Group: Received 2.5% DSS in drinking water and were gavaged with TProtein starting on day 2; (5) DSS + TSPs Group: Received 2.5% DSS in drinking water and were gavaged with TSPs containing EGCG and TProtein starting on day 2. Mice were gavaged with 100 µL solution daily for 8 d.

During the experiment, their environment was kept clean, with free access to food. Body weight, food intake, stool consistency, and rectal bleeding were recorded daily. DAI was calculated to assess colitis severity using the following formula: DAI = (Score for weight loss + Score for stool consistency + Score for rectal bleeding)/3. Weight loss: 0 (none), 1 (1%-5%), 2 (5%-10%), 3 (10%-20%), 4 (>20%). Stool consistency: 0 (normal), 2 (loose), 3-4 (diarrhea). Rectal bleeding: 0 (none), 1 (+), 2 (++), 3 (+++), 4 (++++).

### Behavioral tests

On days 7-8, behavioral tests were conducted, with three mice randomly selected from each group. The open field apparatus consisted of a 50 × 50 cm square arena with walls 30 cm high. Each mouse was placed individually in the center of the arena, and its activity was recorded for 3 min using a video camera. The total distance traveled by the mice during the test was analyzed using SMART 3.0 software. This metric reflects the locomotor activity of the mice, with reduced movement or a preference for staying in the corners indicating depressive-like behavior. Then each mouse was suspended by its tail using adhesive tape fixed 1 cm from the tail's tip. The test lasted for 4 min, during which immobility time was recorded as an indicator of depressive-like behavior. Longer immobility times suggest higher levels of behavioral despair. Mice were placed on a narrow beam (12 mm wide, 50 cm long, elevated 100 cm above the ground) to evaluate their motor coordination and balance. The time taken to traverse the beam and the number of foot slips were recorded.

### Sample collection and analysis

On day 8, mice were first anesthetized, and blood samples were collected via orbital puncture using capillary tubes. The collected blood was allowed to clot at room temperature and then centrifuged at 3000 r/min for 15 min at 4°C to separate the serum, which was stored at -80°C for subsequent biochemical analyses. The mice were then euthanized by cervical dislocation. Spleens were carefully excised, rinsed in sterile saline, and weighed. The entire colon was carefully dissected from the cecum to the rectum and laid flat on a clean surface. The colons were immediately photographed using a high-resolution camera to document macroscopic features, such as length, edema, or hyperemia. The colon length was then measured as an indicator of colitis severity. Colonic contents were collected under sterile conditions, flash-frozen in liquid nitrogen, and stored at -80°C for downstream genomic DNA extraction.

Colon and major organs (heart, liver, spleen, lung, and kidney) were fixed in 4% paraformaldehyde for 24 h and then embedded in paraffin. Tissue sections (5 μm thick) were prepared for H&E staining to evaluate histological damage. For immunofluorescence analysis, paraffin-embedded colon sections were deparaffinized, rehydrated, and subjected to antigen retrieval by heating in a citrate buffer (pH 6.0) for 20 min. After blocking with 5% bovine serum albumin (BSA) for 1 h, the sections were incubated overnight at 4°C with primary antibodies targeting MUC2, Occludin, and ZO-1. After washing with PBS, sections were incubated with fluorescently labeled secondary antibodies for 1 h at room temperature. Fluorescent signals were captured using a fluorescence microscope, and the intensity of staining was quantified using ImageJ software to assess protein expression levels.

The levels of inflammatory cytokines were measured using ELISA kits following the manufacturer's instructions. The colonic contents were subsequently used for 16S rDNA analysis targeting the V3-V4 regions to investigate changes in gut microbiota composition induced by the different treatments. For the above assessments, three samples were randomly selected from each group.

### Statistical analysis

SPSS (2022) was used for the statistical analyses, and results were represented as mean ± SD. Significant differences were confirmed by the ANOVA analysis followed by an LSD post-hoc test with * P < 0.05, ** P < 0.01, *** P < 0.001, ****P < 0.0001, and *****P < 0.00001.

## Supplementary Material

Supplementary figures and table.

## Abbreviations

EGCG: (-)-Epigallocatechin-3-gallate
IBD: Inflammatory bowel disease
ROS: Reactive oxygen species
TSPs: EGCG-loaded tea supraparticles
BBB: Blood-brain barrier
TProtein: Tea proteins
DSS: Dextran sulfate sodium salt
DAI: Disease activity index
UV-vis: Ultraviolet-visible spectroscopy
DLS: Dynamic light scattering
FTIR: Fourier transform infrared spectroscopy
TEM: Transmission electron microscope
DPPH: 1,1-diphenyl-2-picryl-hydrazyl radical
ABTS: 2,2'-azino-bis (3-ethylbenzothiazoline-6-sulfonic acid)
ANOVA: Analysis of variance
LSD: Least significant difference
ELISA: Enzyme-linked immunosorbent assay
H&E: Hematoxylin and Eosin
16S rDNA: 16S ribosomal DNA
PCoA: Principal coordinate analysis
ASVs: Amplicon sequence variants
SCFA: Short-chain fatty acid
LEfSe: Linear discriminant analysis effect size
UPGMA: Unweighted pair-group method with arithmetic means
MUC2: Mucoprotein-2
ZO-1: Zonula occludens-1
FMT: Fecal microbiota transplantation
BSA: Bovine serum albumin
