# Supplementary material for: Tea plant-inspired nanoassembled supraparticles alleviate colitis and associated mental disorders via microbiota-gut-brain interactions
Source: Theranostics. 2025 Jun 18;15(15):7291–307. doi: 10.7150/thno.113573 (PMC12315817; doi:10.7150/thno.113573)
Supplement: Supplementary file 1 — Supplementary figures and table. [file thnov15p7291s1.pdf]

Tea plant-inspired nanoassembled supraparticles alleviate colitis and associated mental disorders via microbiota–gut–brain interactions

Qinling Liu[1,2], Yunxiang He[2], Qingxin Fan[2,3], Yue Wu[2], Jiawen Li[2,4], Siqi Deng[2], Qiuping Xie[2], Yueling Zhao[1], Junling Guo[2,5,6]*, Xiao Du[1]*

[1]Tea Resources Utilization and Quality Testing Key Laboratory of Sichuan Province, College of Horticulture, Sichuan Agricultural University, Chengdu, Sichuan 611130, China.

[2]BMI Center for Biomass Materials and Nanointerfaces, National Engineering Laboratory for Clean Technology of Leather Manufacture, Ministry of Education Key Laboratory of Leather Chemistry and Engineering, College of Biomass Science and Engineering, Sichuan University, Chengdu, Sichuan 610065, China.

[3]Department of Orthopedics, The First Affiliated Hospital of Chongqing Medical University, Chongqing Medical University, Yuzhong, Chongqing 400016, China.

[4]Key Laboratory of Birth Defects and Related Diseases of Women and Children of MOE, Department of Pediatrics, West China Second University Hospital, Sichuan University, Chengdu, Sichuan 610041, China.

[5]State Key Laboratory of Polymer Materials Engineering, Sichuan University, Chengdu, Sichuan 610065, China.

[6]Bioproducts Institute, Department of Chemical and Biological Engineering, The University of British Columbia, Vancouver, BC V6T 1Z4, Canada.

*Corresponding author. E-mail: junling.guo@scu.edu.cn, junling.guo@ubc.ca (J.G.); 10669@sicau.edu.cn (X.D.).

Supplemental Figures and Table

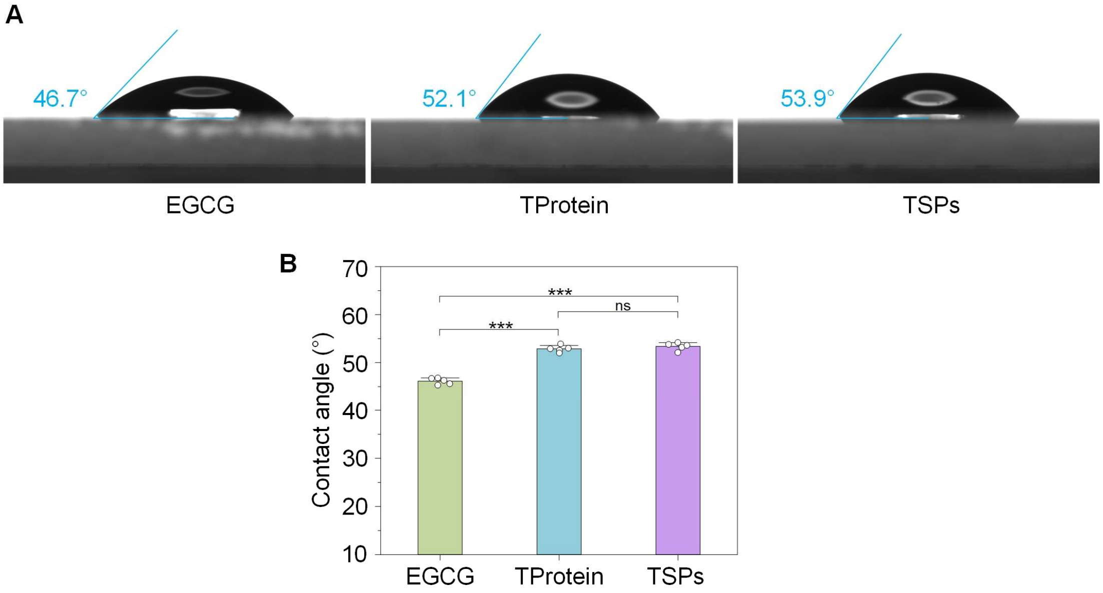

Figure S1. Picture of the contact angle of a droplet on a clean glass (A), and statistical results (B). Data are presented as means ± SD (n = 5). Statistical significance was determined using one-way Analysis of Variance (ANOVA) followed by a least significant difference (LSD) post-hoc test. ns > 0.05, *** P < 0.001.

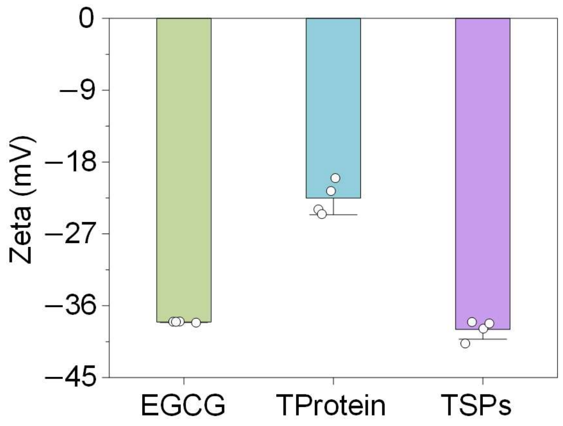

Figure S2. Zeta potential of TSPs. Data are presented as means ± SD (n = 4).

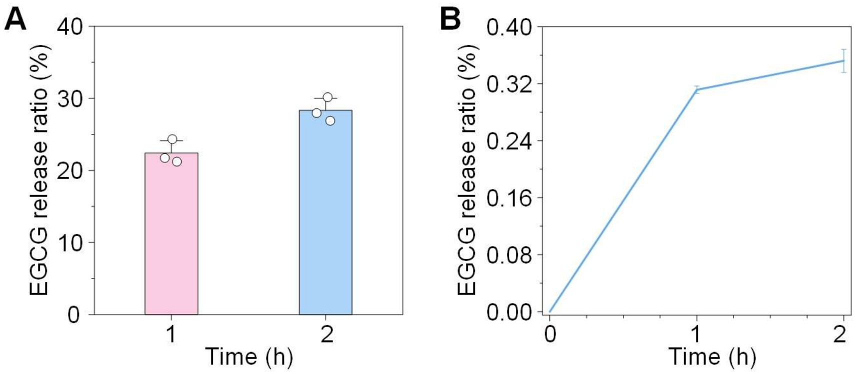

Figure S3. EGCG release from TSPs under simulated gastric conditions. (A) Release ratio of EGCG from TSPs at gastric fluid pH (pH=1.8). When the TSPs solution was adjusted to gastric fluid pH and incubated at 200 rpm for 2 h, less than 30% of EGCG was released. (B) Release of EGCG from freeze-dried TSPs in simulated gastric fluid (pH=1.8). Freeze-dried TSPs incubated in simulated gastric fluid (containing enzymes) for 2 h released less than 40% EGCG. Data are presented as means ± SD (n = 3).

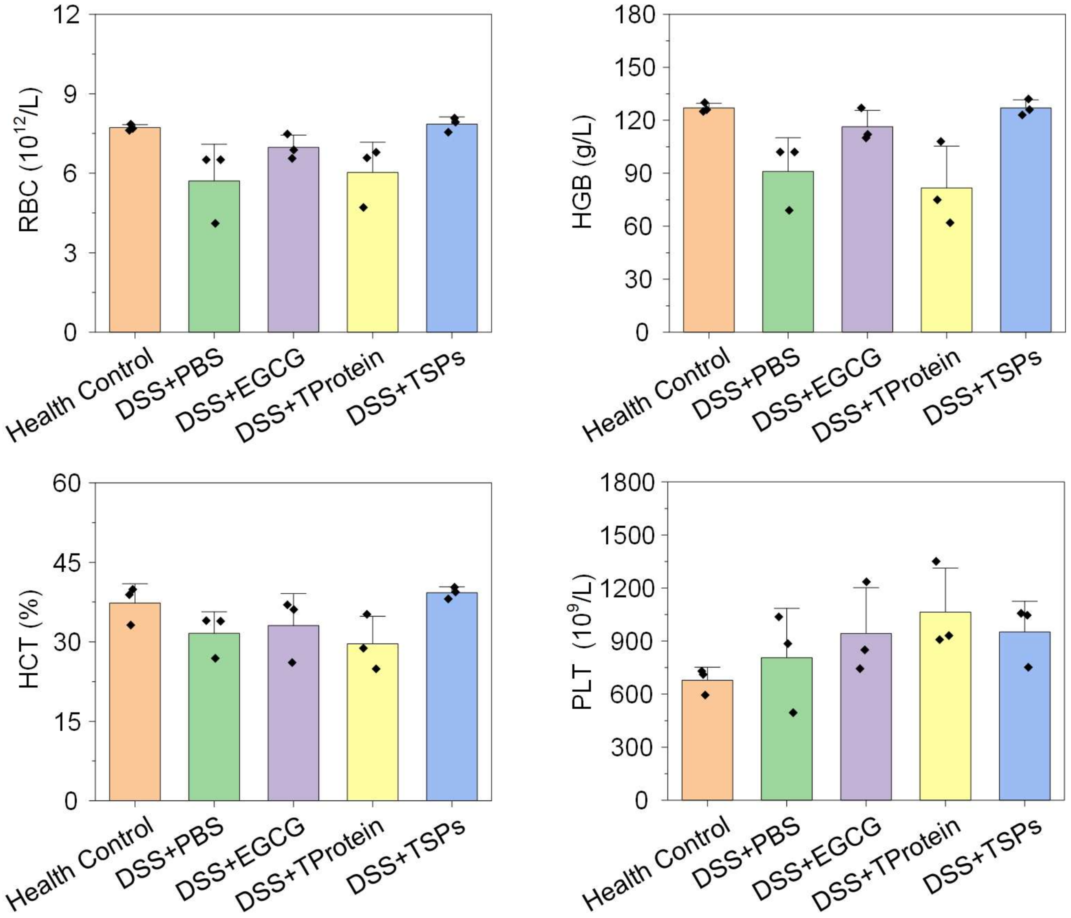

Figure S4. Comparison of red blood cell (RBC) counts, hemoglobin (HGB), hematocrit (HCT), and platelet count (PLT) in peripheral blood. The DSS+PBS group showed signs of iron-deficiency anemia with reduced RBC, HGB, and HCT levels, while TSPs treatment notably improved these parameters, approaching those of the health control group. Data are presented as means ± SD (n = 3). Statistical significance was determined using one-way ANOVA followed by an LSD post-hoc test. ns > 0.05, * P < 0.05.

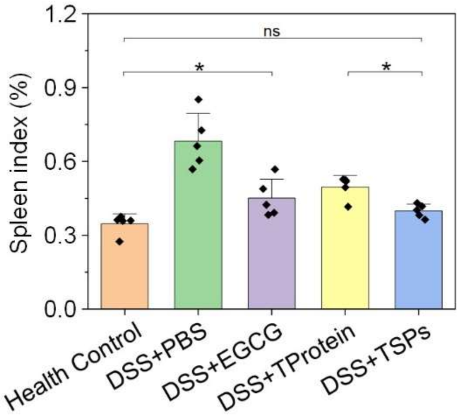

Figure S5. The changes of spleen index in different groups. Data are presented as means ± SD (n = 5). Statistical significance was determined using one-way ANOVA followed by an LSD post-hoc test. ns > 0.05, * $P < 0.05$.

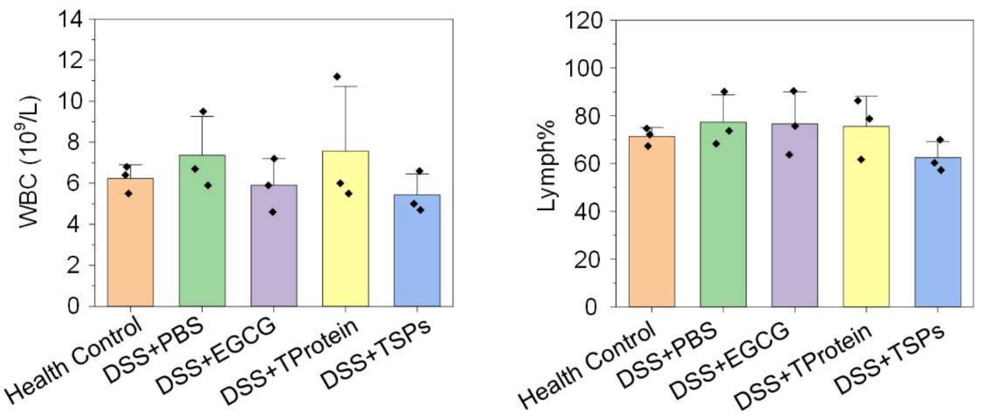

Figure S6. White blood cell (WBC) count and lymphocyte percentage (Lymph%) in peripheral blood from different treatment groups. Data are presented as means ± SD (n = 3).

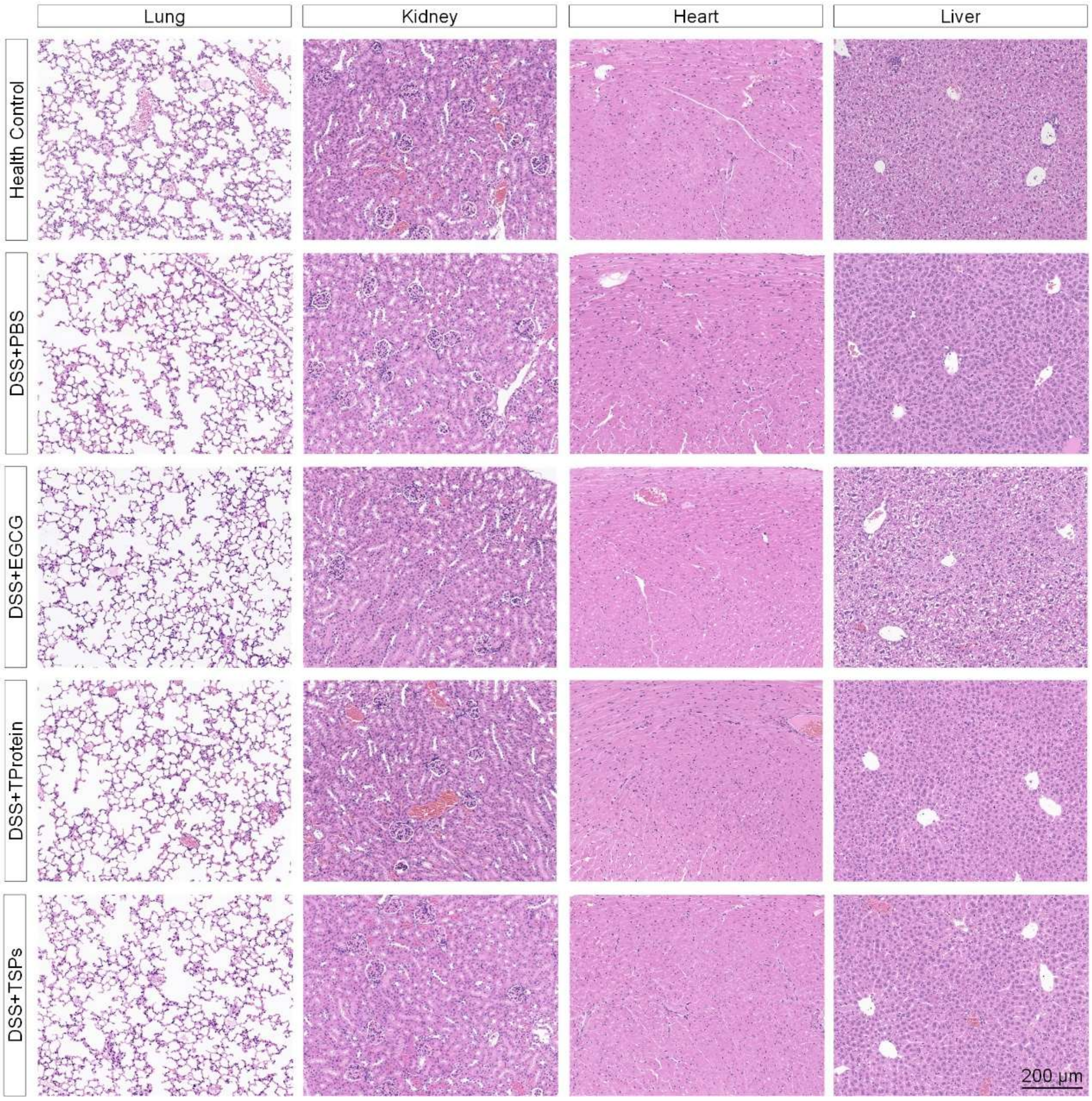

Figure S7. Safety evaluation of TSPs on tissues and organs. H&E staining of the major organs (lung, kidney, heart, and liver) of mice to evaluate the histological differences after different treatments.

Table S1. Secondary structure composition of tea and TProtein.

	α-helix	β-sheet	β-turn	random coli
Tea	9.05 ± 0.91	39.92 ± 0.75	40.57 ± 0.71	10.45 ± 0.47
TProtein	9.31 ± 0.71	34.68 ± 0.42	43.98 ± 0.59	12.03 ± 0.29

Note: Data are presented as means ± SD (n = 3).
